# Anomalies of the Teeth

**Published:** 1844-09

**Authors:** J. R. Dillingham

**Affiliations:** Lynn


*Anomalies of the Teeth.*—Dear Sir—Seeing a number of articles in your Journal in relation to the teeth, I take the liberty to send you a short one on "Anomalies of the Teeth."

The permanent teeth complete their growth at about the age of 21 years. Anomalies, however, sometimes occur—first, in the number of the teeth, and sometimes in an entire deficiency of teeth in the jaw. Mr. Fox mentions a number of cases in which the teeth were entirely deficient. Pliny has mentioned a curious case, which was that of Pyrrhus, king of Epirus. in whom all the crowns were united.

The period at which the teeth appear, varies much. Instances are recorded of children that have been born with teeth. On the other hand, we have heard of instances were the teeth have not appeared for a number of years. A young child, nine and a half months of age, in this town, has twelve teeth —their growth entirely completed. In one instance I inserted four teeth for a lady 40 years of age, two central and two lateral incisors. Three months after they were inserted, she had the plate removed; four months after that time, she had a complete set of natural teeth.

Another case is at present under my observation. A young lady, 22 years old, called on me some months since, and had a cuspid or eye-tooth put in on plate. A few days ago she called at my office, and requested me to fasten the plate in, as it continually got loose. On inspection, I found a new cuspid making its appearance, where it should have been twelve years ago. J. R. Dillingham.

*Lynn, July* 12, 1844.

[*Boston Medical and Surgical Journal.*

